# Supplementary material for: Durvalumab-Associated Pneumonitis in Patients with Locally Advanced Non-Small Cell Lung Cancer: A Real-World Population Study
Source: Curr Oncol. 2023 Dec 9;30(12):10396–407. doi: 10.3390/curroncol30120757 (PMC10742980; doi:10.3390/curroncol30120757)
Supplement: Supplementary file 1 [file curroncol-30-00757-s001.zip › curroncol-2689235-supplementary.pdf]

Table S1. Literature review with large retrospective studies on durvalumab associated pneumonitis; n>50)

Study	Patients who received durvalumab (n)	Pneumonitis (any grade, %)	Severe pneumonitis definition	Severe pneumonitis (%)	Discontinuation from pneumonitis (%)	Corticosteroid use (%)
Current study	189	26	≥ Grade 3	9	In rechallenge, 1 out of 13 patients (8%)	86
Jung et al. 2020 [1]	61	81.0	≥ Grade 3	14.3	In rechallenge, 2 out of 7 patients (28.6%)	N/A
Shaverdian et al. 2020 [2]	62	21	≥ Grade 2	18	48	73
Bruni et al. 2021 [3]	155	17.4	≥ Grade 3	1.9	7.1	N/A
Saito et al. 2021 [4]	225	83	≥ Grade 3	7	In rechallenge, 3 out of 21 patients (14%)	25
Desilets et al. 2021 [5]	147	29.9	≥ Grade 3	6.1	N/A	17
LeClair et al. 2021 [6]	83	25.3	≥ Grade 3	6	67	90
Nishimura et al. 2021 [7]	82	76	≥ Grade 3	5	N/A	n/a
Oshiro et al. 2021 [8]	91	88	≥ Grade 3	12	N/A	68

Tsukita et al. 2021 [9]	87	89	≥ Grade 3	7.4	18.4	N/A
Cruz-Chamorro et al. 2022 [10]	61	37.7	N/A	8.2 (grade 3)	N/A	N/A
Ellison et al. 2023 [11]	78	28.2	Grade 5	9	67	N/A

References

1. Jung, H.A.; Noh, J.M.; Sun, J.-M.; Lee, S.-H.; Ahn, J.S.; Ahn, M.-J.; Pyo, H.; Ahn, Y.C.; Park, K. Real World Data of Durvalumab Consolidation after Chemoradiotherapy in Stage III Non-Small-Cell Lung Cancer. *Lung Cancer* **2020**, *146*, 23–29, doi:10.1016/j.lungcan.2020.05.035.
2. Shaverdian, N.; Thor, M.; Shepherd, A.F.; Offin, M.D.; Jackson, A.; Wu, A.J.; Gelblum, D.Y.; Yorke, E.D.; Simone, C.B.; Chaft, J.E.; et al. Radiation Pneumonitis in Lung Cancer Patients Treated with Chemoradiation plus Durvalumab. *Cancer Med.* **2020**, *9*, 4622–4631, doi:10.1002/cam4.3113.
3. Bruni, A.; Scotti, V.; Borghetti, P.; Vagge, S.; Cozzi, S.; D'Angelo, E.; Giaj Levra, N.; Fozza, A.; Taraborrelli, M.; Piperno, G.; et al. A Real-World, Multicenter, Observational Retrospective Study of Durvalumab After Concomitant or Sequential Chemoradiation for Unresectable Stage III Non-Small Cell Lung Cancer. *Front. Oncol.* **2021**, *11*, 744956, doi:10.3389/fonc.2021.744956.
4. Saito, G.; Oya, Y.; Taniguchi, Y.; Kawachi, H.; Daichi, F.; Matsumoto, H.; Iwasawa, S.; Suzuki, H.; Niitsu, T.; Miyauchi, E.; et al. Real-World Survey of Pneumonitis and Its Impact on Durvalumab Consolidation Therapy in Patients with Non-Small Cell Lung Cancer Who Received Chemoradiotherapy after Durvalumab Approval (HOPE-005/CRIMSON). *Lung Cancer* **2021**, *161*, 86–93, doi:10.1016/j.lungcan.2021.08.019.
5. Desilets, A.; Blanc-Durand, F.; Lau, S.; Hakozaki, T.; Kitadai, R.; Malo, J.; Belkaid, W.; Richard, C.; Messaoudene, M.; Cvetkovic, L.; et al. Durvalumab Therapy Following Chemoradiation Compared with a Historical Cohort Treated with Chemoradiation Alone in Patients with Stage III Non–Small Cell Lung Cancer: A Real-World Multicentre Study. *Eur. J. Cancer* **2021**, *142*, 83–91, doi:10.1016/j.ejca.2020.10.008.
6. LeClair, J.N.; Merl, M.Y.; Cohenuram, M.; Luon, D. Real-World Incidence of Pneumonitis in Patients Receiving Durvalumab. *Clin. Lung Cancer* **2022**, *23*, 34–42, doi:10.1016/j.cllc.2021.08.006.
7. Nishimura, A.; Ono, A.; Wakuda, K.; Kawabata, T.; Yabe, M.; Miyawaki, T.; Miyawaki, E.; Kodama, H.; Nishioka, N.; Mamesaya, N.; et al. Prognostic Impact of Pneumonitis after Durvalumab Therapy in Patients with Locally Advanced Non-Small Cell Lung Cancer. *Invest. New Drugs* **2022**, *40*, 403–410, doi:10.1007/s10637-021-01191-6.
8. Oshiro, Y.; Mizumoto, M.; Sekino, Y.; Maruo, K.; Ishida, T.; Sumiya, T.; Nakamura, M.; Ohkawa, A.; Takizawa, D.; Okumura, T.; et al. Risk Factor of Pneumonitis on Dose-Volume Relationship for Chemoradiotherapy with Durvalumab: Multi-Institutional Research in Japan. *Clin. Transl. Radiat. Oncol.* **2021**, *29*, 54–59, doi:10.1016/j.ctro.2021.05.009.
9. Tsukita, Y.; Yamamoto, T.; Mayahara, H.; Hata, A.; Takeda, Y.; Nakayama, H.; Tanaka, S.; Uchida, J.; Usui, K.; Toyoda, T.; et al. Intensity-Modulated Radiation Therapy with Concurrent Chemotherapy Followed by Durvalumab for Stage III Non-Small Cell Lung Cancer: A Multi-Center Retrospective Study. *Radiother. Oncol.* **2021**, *160*, 266–272, doi:10.1016/j.radonc.2021.05.016.
10. Cruz-Chamorro, R.J.; Rishi, A.; Liveringhouse, C.; Bryant, J.M.M.; Perez, B.A.; Rosenberg, S.A.; Dilling, T.J. Real World Rates and Predictive Factors of Pneumonitis in Advanced, Non-Resectable NSCLC Treated with Concurrent Chemoradiation and Durvalumab. *Int. J. Radiat. Oncol.* **2022**, *114*, e370, doi:10.1016/j.ijrobp.2022.07.1502.
11. Ellison, C.; Martens, M.; Alvarez Argote, J.; Benz, S.; Currey, A.; Johnstone, C.; Klawikowski, S.; Livingston, K.; Longo, J.M.; Menon, S.; et al. High-Grade Pneumonitis Events in Unresectable, Locally Advanced Non-Small Cell Lung Cancer Patients Treated with Definitive Chemoradiation Followed by Adjuvant Durvalumab. *JTO Clin. Res. Rep.* **2023**, 100537, doi:10.1016/j.jtocrr.2023.100537.
